# Efficacy and safety of Zhishixiaopi decoction in functional dyspepsia: A meta-analysis of randomized controlled trials

**DOI:** 10.1371/journal.pone.0301686

**Published:** 2024-05-29

**Authors:** Xiankun Zhao, Xinyu Cheng, Jing Ye, Jiaqing Ren, Bin Li, Dongmei Tan, Tangshan Li, Kai Zhou, Jing Pu, Xia Luo, Yong Feng

**Affiliations:** 1 Department of Integrated Traditional Chinese and Western Medicine, Mianyang Central Hospital, School of Medicine, University of Electronic Science and Technology of China, Mianyang, Sichuan Province, China; 2 Grand Central Pain Relief, New York, NY, United States of America; 3 Department of Rehabilitation Medicine, West China Second University Hospital of Sichuan University, Chengdu, Sichuan Province, China; 4 Liucheng Street Community Health Service Center, Chengdu, Sichuan Province, China; The Technical University of Kenya, KENYA

## Abstract

**Background:**

Functional dyspepsia (FD) refers to a group of clinical symptoms caused by gastric and duodenal dysfunction. Which is a chronic functional disorder of the gastrointestinal tract with no cure. Zhishixiaopi decoction (ZSXP) is a type of Chinese herbal prescription that for treating FD. Although some randomized controlled trials (RCTs) report that ZSXP can significantly improve FD clinical symptoms and/or laboratory results, the trial design varies greatly among studies, making it challenging to draw a conclusion of the efficacy of ZSXP in treating FD.

**Design:**

A systematic review and a meta-analysis.

**Setting:**

Mianyang Central Hospital.

**Objective:**

We conducted a systematic review and a meta-analysis to evaluate the efficacy and safety of ZSXP for treating FD.

**Methods:**

We developed inclusion and exclusion criteria based on FD diagnosed criteria, interventions to treat FD, and outcomes of these interventions. Search strategies combined disease terms, symptom terms, anatomy terms and intervention terms. Literature search was conducted on eight online databases in English or Chinese, including Medline (via PubMed), Embase (via Ovid), The Cochrane Library, Web of Science, China Biology Medicine (CBM), China National Knowledge Infrastructure (CNKI), Chinese Scientific Journals Database (VIP), and Wanfang Database.

**Intervention:**

The experimental group received oral administration of ZSXP and had a complete treatment process. ZSXP needs to fully contain the key herbal ingredients, regardless of whether the dosage of each herb is consistent with the original prescription. The Control group received monotherapy or combination therapy of other Western medicine and had a complete treatment process.

**Outcomes:**

The primary outcomes appraised were Total effective rate (TER), serum levels of Motilin(MOT), Gastrin(GAS) and Somatostatin (SS), Gastric emptying rate (GER) using a Barium meal method (GER(B)) and Gastric half emptying time using an Ultrasonic method (GHET(T_1/2_)). The Cochrane Bias Risk Tool was used for quality critical appraisal, Review Manager (RevMan) version 5.3 was used for statistical analysis.

**Results:**

A total of 21 medium-quality RCTs were included in the meta-analysis. All 21 included studies were conducted and completed in Mainland China from 1998 to 2020. The treatment duration was between two weeks to two months. The meta-analysis suggests that, compared with the Western medicine treatment group, ZSXP treatment was more effective to improving the TER in FD [Odds ratio, OR = 3.54, 95%CI:(2.49, 5.05), *Z* = 6.99, P<0.00001] without significant increase in adverse events. However, no statistical significance was found between the groups in serum MOT levels [Standard mean difference, SMD = 1.05, 95%CI:(-0.42, 2.53), *Z* = 1.04, *P* = 0.16], serum GAS levels [SMD = -0.16, 95%CI:(-1.20, 0.88), *Z* = 0.31, *P* = 0.76], serum SS levels [SMD = -0.04, 95%CI:(-1.97, 1.89), *Z* = 0.04, *P* = 0.97], GER(B) [SMD = 1.09, 95%CI:(-0.81, 3.00), *Z* = 1.12, *P* = 0.26]or GHET(T1/2) [Mean difference, MD = -2.18, 95%CI:(-5.55, 1.19), *Z* = 1.27, *P* = 0.20].

**Conclusions:**

The meta-analysis suggests that Zhishixiaopi treatment is a relatively effective and safe traditional Chinese medicine prescription and could be used for functional dyspepsia treatment. Considering the limitations of this study, the conclusion needs to be further confirmed by high-quality, multi-center, and large-sample randomized controlled trials.

## Introduction

Functional (non-ulcer) dyspepsia (FD) refers to a group of clinical symptoms caused by gastric and duodenal dysfunction. The organic diseases causing these symptoms are excluded by examination and not counted as FD. According to the Rome IV classification [1], The condition is subdivided into three categories postprandial distress syndrome (PDS), epigastric pain syndrome (EPS), and overlap among EPS and PDS. FD symptoms include upper abdominal pain, burning sensation in the upper abdomen, fullness after a meal, and early satiety. These symptoms may be accompanied by other symptoms such as upper abdominal distension, belching, loss of appetite, nausea, and vomiting. FD is one of the common gastrointestinal conditions that affect almost 1/5th of the population [1]. The prevalence of FD is approximately 21.8% globally [2], 10% in the UK [3] and 8% to 23% in Asia [4]. Risk factors include psychological comorbidity, acute gastroenteritis, female sex, smoking, use of non-steroidal anti-inflammatory drugs, and Helicobacter pylori infection [2]. FD is a chronic functional disorder of the gastrointestinal tract with no cure [2], Therefore, FD has an impact on daily work, quality of life and mental health [5], and has brought a huge economic burden to the healthcare system [6]. The economic impact is estimated to be over US$18 billion per year in the USA [2].

Currently, with its unclear pathophysiological mechanisms, FD is diagnosed by clinical symptoms and no accurate biological markers are found. One possible pathogenesis of FD is brain-gut axis malfunction [2], such that disturbance of brain-gut peptide (BGP) levels in the brain-gut axis leads to the occurrence of FD. BGP is a small molecular peptide substance distributed among the central nervous system (CNS), enteric nervous system (ENS), and endocrine cells of the gastrointestinal tract. Motilin (MOT), Gastrin (GAS), and Somatostatin (SS) all belong to BGP small molecules. MOT is a gastrointestinal (GI) peptide hormone synthesized and released by enterochromaffin cells in the proximal small intestine [7]. MOT is a stimulus for strong antral contractions and has a hunger signaling function. It stimulates proximal gastrointestinal (GI) motility [8–10] and is involved in the regulation of upper GI motility [11]. GAS is released by G-cells in the stomach and is a major stimulus for gastric acid secretion [12]. SS is released in the stomach as well as the small bowel and has a strong inhibitory effect on GI motility and secretion [12]. The higher level of SS was often correlated with a higher symptom burden of FD [13]. SS levels are also correlated with heartburn severity scores [14]. Thus, MOT, GAS and SS are usually used as detection indicators of brain-gut axis function and measures to reflect FD GI motility in randomized controlled trials (RCTs).

Additionally, gastric emptying rate (GER) is an indicator that can reflect gastrointestinal function. Previous studies have shown that a substantial percentage of FD patients have gastric emptying delays [15] and that up to 25%-30% of FD [16, 17] patients show impaired gastric emptying. Also, GER is usually used as a detection indicator to reflect gastrointestinal function and indicate symptom relief of FD in RCTs.

The treatment of FD is still based on symptomatic relief. Western medicine remains one of the most popular treatment methods, including eradication of Helicobacter pylori (if the infection is present), proton pump inhibitors, histamine-2 receptor antagonists, prokinetics and central neuromodulators [2]. Prokinetic drugs are the most commonly used drugs. Unfortunately, monotherapy of these drugs is ineffective in resolving a series of clinical symptoms of FD and often accompanied with side effects [18, 19]. Therefore, the interest in treatment using alternative medicine has been increasing [50].

Chinese herbal medicine (CHM) has been used as an alternative treatment in the Asia-Pacific region for thousands of years. Previous studies suggested that CHM treatment has great potential and safety in alleviating the symptoms of FD [20–23]. In particular, Zhishixiaopi decoction (ZSXP) is a Chinese herbal medicine prescription from “Lanshi Micang” (Jin Dynasty. Li Dongyuan), a book documenting Chinese herbal medicine prescriptions deemed effective through clinical practice [24]. As recorded in the original book: Shixiao Pill (also named Zhishi Xiaopi Pill), is used to treat stringy pulse on chi (cun, guan and chi, three places at the wrist where the pulse is usually taken), deficiency of the lower heart (ancient anatomical location, usually referring to the stomach), "Pi Man" [24]. ZSXP can appetize or promote eating and is used to treat discomfort in the stomach, aversion to food, laziness, tiredness, bloating and fullness symptoms of FD [24]. ZSXP is mainly composed of Zhishi (dried young fruit of *Citrus aurantium* L. or C. *sinensis* Osbeck.), Houpo (bark of *Magnolia officinalis* Rehd. et Wils or *M*. *officinalis* Rehd. et Wils. var. *biloba* Rehd. et Wils.), Huanglian (dried root of *Coptis chinensis* Franch, C. *deltoidea* C. Y. Cheng et Hsiao or C. *teeta* Wall.), Banxia (tubers of *Pinellia ternate* (Thunb.) Breit.), Ganjiang (dried tubers of *Zingiber officinale* Rosc.), Maiya (dried product of *Hordeum vulgare* L. ’s ripe fruit after germination), Renshen (root of *Panax ginseng* C. A. Mey.), Baizhu (root of *Atractylodes macrocephala* Koidz.), Fuling (sclerotia of *Poria cocos*(Schw.) Wolf) and Zhigancao (roasted roots and roasted rhizomes of *Glcyrrhiza uralensis* Fisch., *G*. *inflata* Bat. or *G*. *glabra* L.) [25] (Table 1).

**Table 1:** Herbal components in ZSXP.

No	Name	Dose(gram)
1	Zhishi (dried young fruit of *Citrus aurantium* L. or C. *sinensis* Osbeck.)	15
2	Houpo (bark of *Magnolia officinalis* Rehd. et Wils or *M*. *officinalis* Rehd. et Wils. var. *biloba* Rehd. et Wils.)	12
3	Huanglian (dried root of *Coptis chinensis* Franch, C. *deltoidea* C. Y. Cheng et Hsiao or C. *teeta* Wall.)	15
4	Banxia (tubers of *Pinellia ternate* (Thunb.) Breit.)	12
5	Ganjiang (dried tubers of *Zingiber officinale* Rosc.)	6
6	Maiya (dried product of *Hordeum vulgare* L. ’s ripe fruit after germination)	6
7	Renshen (root of *Panax ginseng* C. A. Mey.)	12
8	Baizhu (root of *Atractylodes macrocephala* Koidz.)	6
9	Fuling (sclerotia of *Poria cocos*(Schw.) Wolf)	6
10	Zhigancao (roasted roots and roasted rhizomes of *Glcyrrhiza uralensis* Fisch., *G*. *inflata* Bat. or *G*. *glabra* L.)	6

In recent years, RCTs have shown that ZSXP can significantly improve the clinical symptoms and laboratory results of FD. For example, a study suggests that the effect of ZSXP on enhancing gastric motility is comparable with but no better than that of Cisapride [26]. In another RCT, ZSXP combined with prokinetic drugs shows clinical effect on treating FD and can effectively improve the level of gastrointestinal hormone [27]. ZSXP treatment is also comparable to Mosapride citrate tablet in the treatment of FD [28]. Some studies suggest that ZSXP has adverse events, including dizziness, rash and fatigue [29]. However, the quality of these studies varies, necessitating a systematic review and a meta-analysis to reliably assess the efficacy and safety of ZSXP for FD in clinical practice.

## Methods

### Ethics statement

As all analyses were based on previously published studies, no Institutional Review Board approval or patient consent was required.

### Design

A systematic review and a meta-analysis.

### Setting

Mianyang Central Hospital.

### Protocol and registration

The meta-analysis was performed and reported in accordance with the Preferred Reporting Items for Systematic Reviews and Meta Analyses (PRISMA) guidelines (see S1 Checklist). The protocol was preregistered (systematic review registration: http://www.crd.york.ac.uk/PROSPERO. PROSPERO registration number: CRD42021292116).

### Literature search

The sources of Medline (via PubMed), Embase (via Ovid), The Cochrane Library, Web of Science, China Biology Medicine (CBM), China National Knowledge Infrastructure (CNKI), Chinese Scientific Journals Database (VIP), and Wanfang Database were comprehensively searched using the creation of publication date between the creation of the database and January 8, 2023. The search terms included combined disease terms (e.g. indigestion, dyspepsia, disturbance, and dysfunction), symptom terms (e.g. burning, pain, discomfort, discomfort, and distress), anatomy terms (e.g. epigastric, gastro, stomach, intestinal, digest) and intervention terms (e.g. Zhishixiaopi* OR Zhishi Xiaopi* OR Zhi shi xiao pi* OR Kaiwei* OR Shixiaowan in Chinese). The MeSH keywords are "Herbal medicine", "Plants, medicinal", "Medicine, traditional", and "Drugs, Chinese herbal". No language restrictions were applied. Unpublished studies were not included. Detailed search strategy for each database can be found in Supplementary Materials (see S1 File).

### Inclusion criteria

Studies that met all the following criteria were included: (1) The individuals were diagnosed of FD according to any recognized criteria (including but not limited to RIIC: Rome II Criteria and RIIIC: Rome III Criteria). The inclusion criteria did not concern age, gender, or race. (2) Experimental group received oral administration of ZSXP and had a complete treatment process. ZSXP needs to fully contain the above ten key herbal ingredients, regardless of whether the dosage of each herb is consistent with the original prescription. (3) Control group received monotherapy or combination therapy of other Western medicine and had a complete treatment process. (4) Outcome reported the symptom score or laboratory test results. The FD symptom score relief of 30% and above is considered as effective. (5) Study was designed as randomized clinical controlled trail.

### Exclusion criteria

Studies that met any of the following criteria were excluded: (1) The study has unclear diagnosis criteria for FD or the data includes other types of dyspepsia (e.g., dyspepsia induced by drugs or caused by secondary pathologies and Irritable Bowel Syndrome (IBS])). (2) Experimental group included other interventions other than ZSXP, or ZSXP was not orally administrated. (3) Control group received other unidentified medicine. (4) There are no available statistical findings. (5) The study is a case report, review, animal or non-clinical study, duplicate publications, unverified RCTs, or with no or inappropriate control group.

### Study selection

We conducted a single literature search and paper selection on the inclusion and exclusion criteria above. Two researchers independently review the same search output list. First, each researcher searched the databases and read titles, abstracts and keywords to determine their appropriateness. Next, they browsed remaining articles and screen them. It should be noted that to cover the target literature as much as possible, the search strategy includes a combination of symptom items rather than only FD diagnosis. Considering the overlap of symptoms between FD and other diseases (e.g., IBS, drug-induced secondary constipation, etc.), it is required that recognized FD diagnostic criteria is mentioned in the full text to ensure that all patients are diagnosed of FD. Finally, articles were screened again based on the inclusion and exclusion criteria, and the selected articles were extracted for meta-analysis. For each selected article, the following data were recorded: authors’ names, year of publication, diagnostic criteria, duration of treatment, statistics of age, sample size, intervention methods, outcome measures (including symptom improvement and other outcomes) and adverse events. Data were independently extracted as intention-to-treat analyses (ITT analyses) by two researchers, drop-outs were assumed as treatment failures in experimental groups and as efficiency in control groups in ITT. Data were repeatedly confirmed and checked for internal consistency. In case there was disagreement between the two researchers, a third researcher was consulted. Cohen’s Kappa was used to assess the concordance between the two independent reviewers.

### Outcome categorization

The degree of FD symptom relief was evaluated by the Total effective rate (TER). Specifically, we first calculated Efficacy index using the formula: Efficacy index = (pre-treatment symptom score–post-treatment symptom score)/ pre-treatment symptom score. An Efficacy index≥30% was defined as effective. TER was then computed as number of effective individuals/sample size. Laboratory tests (serum levels of MOT, GAS and SS, Gastric emptying rate (GER) using a Barium meal method (GER(B)) and Gastric half emptying time using an Ultrasonic method (GHET(T_1/2_)) were reported as objective outcome indicators in primary studies.

### Quality critical appraisal

The Cochrane Bias Risk Tool was used to evaluate the quality of the included articles to ensure the reliability of the final meta-analysis results. Bias types mainly include selection bias, performance bias, detection bias, attritions bias, reporting bias and other biases. The results of each study were judged according to the following criteria: “yes” (low risk of bias), “no” (high risk of bias), or “unclear” (uncertain risk of bias). Quality assessments were performed independently by two researchers, followed by cross-validation between the researchers.

### Statistical analysis

Review Manager (RevMan) version 5.3 (Cochrane Collaboration) software was used for statistical analysis. Dichotomous data or count data were tested using odds ratio (OR). Continuous data were analyzed by testing mean difference (MD) or standard mean difference (SMD). For each test, its corresponding 95% confidence interval (CI) was calculated. *P*<0.05 was considered statistically significant. Χ^2^ test and I^2^ test were conducted to assess the statistical heterogeneity between studies. When evidence for homogeneity (P>0.1 or I^2^<50%) was present, a fixed-effects model was used. Conversely, when there was sign of heterogeneity (P<0.1 or I^2^>50%), a random-effects model was used. For significant heterogeneity, sensitivity analyses were performed to assess the stability of this study and, when necessary, subgroup analyses were performed to explore the sources of this heterogeneity.

## Result

### Literature search

**Fig 1:**
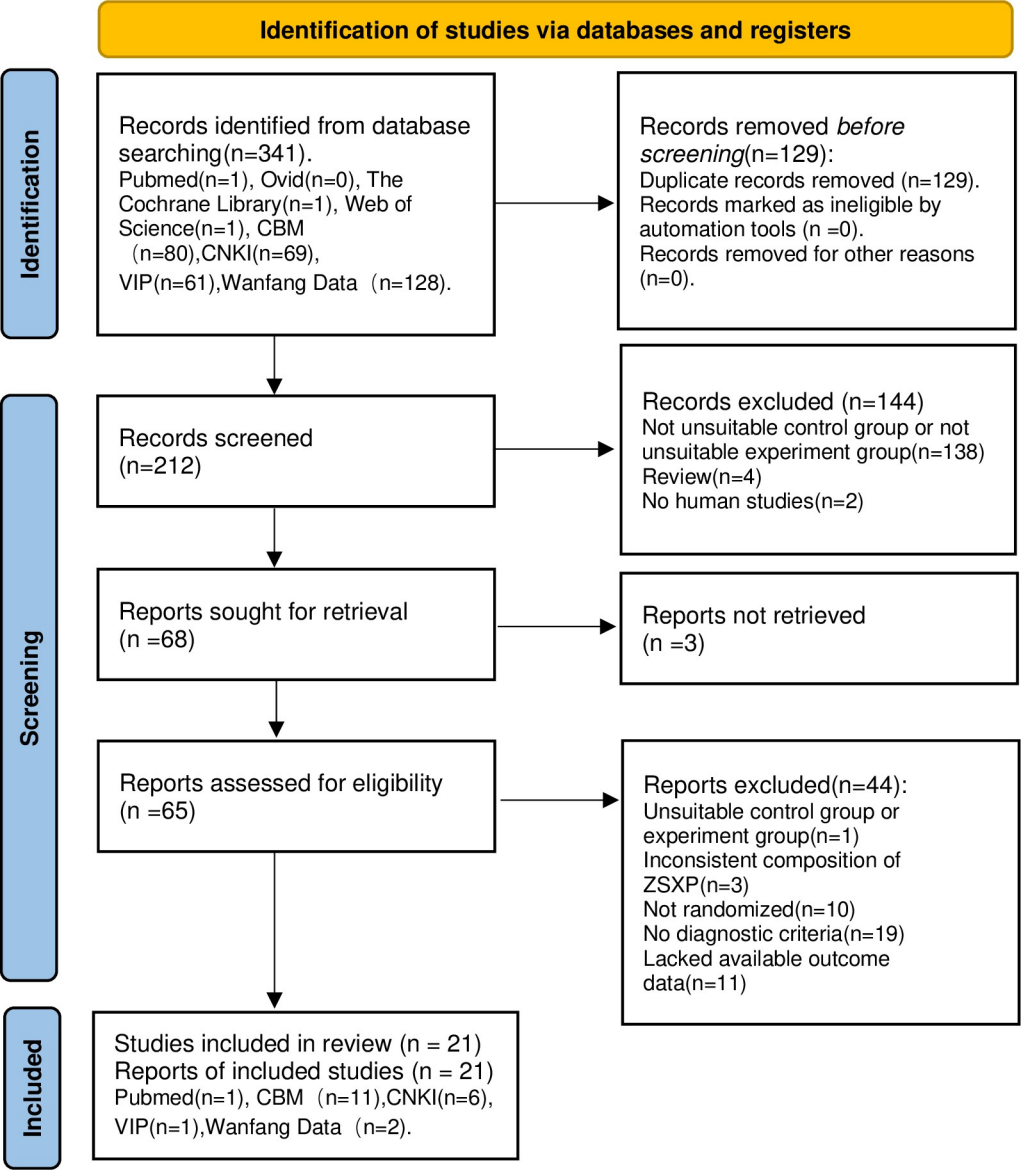
PRISMA 2020 flow diagram. Study selection process for the meta-analysis with exclusion criteria noted.

According to the pre-registered search strategy, a total of 341 articles were retrieved. Among them, Pubmed, The Cochrane Library, Web of Science each had 1 search result. Ovid had 0 search result. and 80 search results for CBM, 69 for CNKI 61 for VIP, 128 for Wanfang Data. Then, full-text preview of 65 articles was conducted. Finally, 21 studies [26–46] were included in the meta-analysis (Fig 1). Cohen’s Kappa was used to assess the concordance between the two independent reviewers. The kappa score of 0.9 (*k*≈0.9) indicates almost perfect agreement between the two reviewers (details can be found in **S2 File**).

### Methodological quality of studies included

**Fig 2:**
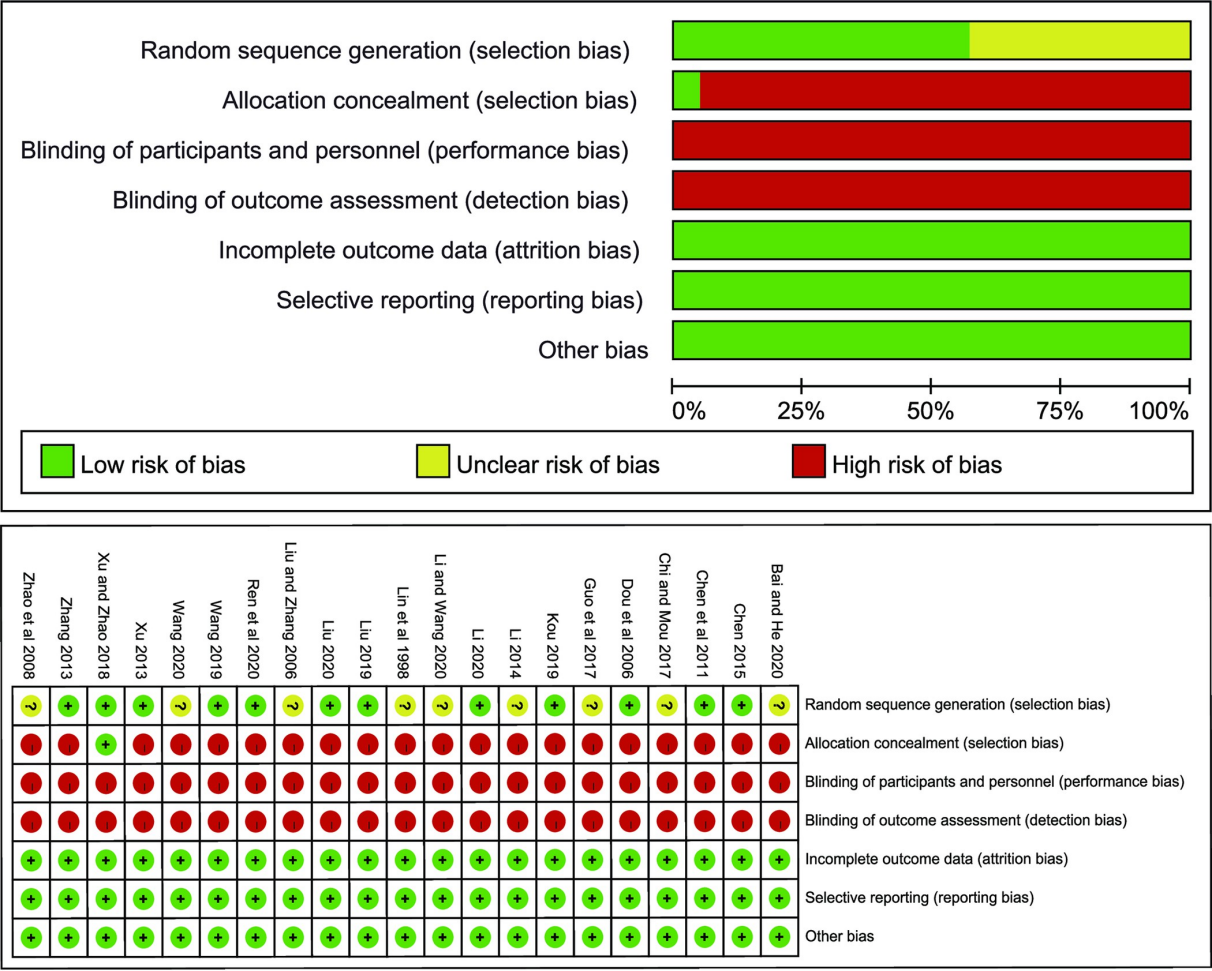
Risk of bias graph for all included studies.

The Cochrane Bias Risk Tool was used to evaluate the methodological quality of the included studies. All the studies mentioned randomization, twelve studies [27–30, 32, 36–39, 41, 45, 46] recorded specific randomization methods. Randomization method of 9 studies [26, 31, 33–35, 40, 42–44] was unknown. Only one study [29] used the envelope method to allocate concealment, thus the selection bias of other studies was high. None of the studies used blinding, thus the performance bias and detection bias of all studies were high. All studies have complete data, thus have low attrition bias, low reporting bias and low other bias. In general, all included studies were of medium quality. More detailed methodological quality assessment results can be seen in Fig 2 (Fig 2).

### Study characteristics

All 21 included studies [26–46] were conducted and completed in Mainland China from 1998 to 2020. Twenty studies [27–46] are published in Chinese, and only one study [26] is published in English in an international journal. The diagnostic criteria of 18 studies are the Roman criteria, and the diagnostic criteria of one study [36] are based on Talley et al.,1999 [47]. The treatment duration was between two weeks to two months. Except for Xu and Zhao, 2018 [29] that uses children as participants, all of the rest of the studies recruit adults as participants. The sample size of a group ranges from 24 to 134 subjects. Except for Dou et al., 2006 [36] and Guo et al., 2017 [35], the other nineteen studies have two groups. Dou et al., 2006 [36] is a four-arm design, and Guo et al 2017 [35] employs a three-arm design. Ten studies [26, 28, 30–36, 41] test the monotherapy efficacy of ZSXP against Western medicine. In the ten studies, nine [26, 28, 30–36] use prokinetic drugs in the control groups (Mosapride [28, 30–33, 35], Domperidone [34], and Cisapride [26, 36]). Four studies [37–40] test the efficacy of ZSXP monotherapy against the multi-therapy of Western medicine. Four studies [29, 42–44] test the efficacy of ZSXP multi-therapy against monotherapy of Western medicine. Three studies [27, 45, 46] test the efficacy of ZSXP multi-therapy against other Western medicine multi-therapy. A total of thirteen studies [28, 30–34, 37, 39–44] report TER, and twelve studies [26, 27, 29, 34–39, 42, 45] document laboratory test results. Eleven studies [28–34, 37, 39, 41, 44] record adverse events. Detailed information can be found in Table 2 (Table 2).

**Table 2:** Characteristics of the included studies in meta-analysis.

Study	Diagnostic criteria	Treatment Duration	Age (years±SD) by group	Sample Size	Interventions	Outcome measures		Adverse event
						Symptom improvement (TER)	Others	
**Chen et al., 2011 [30]**	②	4 **W**	**E**:42.30±6.23 **C**:41.20±6.41	30/30	**E**:ZSXP(BID) **C**:Mosapride(5mg, TID)	**E**:83.33%(25/30) **C**:60.00%(18/30)	NR	Included
**Chen, 2015 [28]**	③	15 **D**	**E**:48.63±9.66 **C**:47.26±9.23	30/30	**E**:ZSXP(BID) **C**:Mosapride(5mg, TID)	**E**:86.67%(26/30) **C**:83.33%(25/30)	NR	Included
**Li, 2014 [31]**	③	4 **W**	**E**:42.9±13.5 **C**:42.7±12.3	28/28	**E**:ZSXP(BID) **C**:Mosapride(5mg, TID)	**E**:96.43%(27/28) **C**:89.29%(25/28)	NR	Included
**Liu, 2019 [32]**	④	4 **W**	**E**:45.40±10.13 **C**:44.63±10.58	30/30	**E**:ZSXP(BID) **C**:Mosapride(5mg, TID)	**E**:93.33%(28/30) **C**:73.33%(22/30)	NR	Included
**Wang, 2020 [33]**	④	4 **W**	**E**:45.60±11.17 **C**:45.69±10.32	42/42	**E**:ZSXP(BID) C:Mosapride(5mg, TID)	**E**:88.09%(37/42) **C**:64.29%(27/42)	NR	Included
**Zhao et al., 2008 [34]**	③	2 **W**	**E**:18–65 **C**:20–65	36/32	**E**:ZSXP(TID) **C**:Domperidone(10mg,TID)	**E**:94.46%(34/36) **C**:81.25%(26/32)	GHET (T_1/2_)	Included
**Guo et al., 2017 [35]**	③	2 **W**	**E1**:51.2±6.14 **E2**:52.9±4.12 **C**:52.2±6.29	24/25/25	**E1**:ZSXP(BID) **E2**:ZSXP(BID)+Mosapride(5 mg, TID) **C**:Mosapride(5mg, TID)	NA	MOT/GAS/ SS/GER(B)	NR
**Lin et al., 1998 [26]**	①	4 **W**	**E**:45.1±9.67 **C**:48.5±12.9	27/24	**E**:ZSXP(TID) **C**:Cisapride(5mg,TID)	NA	GHET(T_1/2_)	NR
**Dou et al., 2006 [36]**	⑦	4 **W**	**E1**:39.76±12.12 **E2**:37.27±10.29 **E3**:35.22±11.35 **C**:36.30±11.29	66/ 46/ 45/ 42	**E1**:ZSXP(BID)Chinese Herbal Pieces Group **E2**:ZSXP(BID)Combined decoction granule Group **E3**:ZSXP(BID)Separated decoction granule group **C**:Cisapride(5mg,TID)	NA	MOT/ EM/ VNTM	NR
**Xu, 2013 [37]**	③	4 **W**	**E**:40.68±11.41 **C**:40.31±12.78	66/63	**E**:ZSXP(BID) **C**:Domperidone(10mg,TID)+Magnesium aluminum carbonate (1.0g,TID)	**E**:83.33%(55/66) **C**:61.90%(39/63)	GER(B)	Included
**Zhang, 2013 [38]**	③	4 **W**	E:38.80±11.79 C:38.86±12.31	66/63	**E**:ZSXP(BID) **C**:Domperidone(10mg,TID)+Magnesium aluminum carbonate (1.0g,TID)	NA	VIP, LEP	NR
**Kou, 2019 [39]**	①	1 **M**	**E**:42.85±6.04 **C**:41.14±5.74	80/80	**E**:ZSXP(BID) **C**:Domperidone(10mg,TID)+Omeprazole(20mg,BID)	**E**:88.75%(71/80) **C**:71.25%(57/80)	SF-36/SAS	Included
**Chi and Mou, 2017 [40]**	③	4 **W**	**E**:53.6±6.25 **C**:52.3±7.12	35/35	**E**:ZSXP(BID) **C**:Pantoprazole(40mg, QD)+Mosapride(5mg, TID)	**E**:94.29%(33/35) **C**:82.86%(29/35)	NR	NR
**Li, 2020 [41]**	④	4 **W**	**E**:47.86±9.01 **C**:48.78±9.69	28/27	**E**:ZSXP(BID) **C**:Azintamide(150mg, TID)	**E**:92.9%(26/28) **C**:70.4%(19/27)	NR	Included
**Xu and Zhao, 2018 [29]**	⑤	2 **W**	**E**:9.17±1.82 **C**:9.23±1.84	134/134	**E**:ZSXP pills(0.1g/Kg,TID) +Mosapride(5mg, TID) **C**:Mosapride(5mg, TID)	NA	GER(U)	Included
**Bai and He, 2020 [42]**	④	2 **W**	**E**:45.16±3.51 **C**:44.25±3.68	53/53	**E**:ZSXP(NR)+Mosapride(5 mg, TID) **C**:Mosapride(5mg, TID)	**E**:96.23%(51/53) **C**:84.91%(45/53)	MOT/GAS/ GMFI/SF-36/ GHET (T_1/2_)	NR
**Liu and Zhang, 2006 [43]**	⑥	4 **W**	**E**:57±23.5 **C**:55±25.1	50/50	**E**:ZSXP(QD)+ Mosapride(5mg, TID) **C**:Mosapride(5mg, TID)	**E**:94.00%(47/50) **C**:76.00%(38/50)	NR	NR
**Li and Wang, 2020 [44]**	④	4 **W**	**E**:42.5±3.1 **C**:41.2±3.1	45/45	**E**:ZSXP(BID)+Azintamide(150mg, TID) **C**:Azintamide(150mg, TID)	**E**:95.6%(43/45) **C**:82.2%(37/45)	NR	NR
**Liu, 2020 [45]**	③	4 **W**	**E**:54.01±6.25 **C**:53.12±6.24	56/56	**E**:ZSXP(BID)+Domperidone(10mg,TID)+Omeprazole(20mg, BID) **C**:Domperidone(10mg,TID)+Omeprazole(20mg, BID)	NA	MOT/GAS/ SS/GER(B)	Included
**Ren et al., 2020 [27]**	③	2 **M**	**E**:33.2±8.5 **C**:32.1±8.2	44/44	**E**:ZSXP pills(6g,TID)+Trimebutine(0.2g TID) **C**:Trimebutine(0.2g TID)	NA	HP/5-HT/ SS/NO	NR
**Wang, 2019 [46]**	③	1 **M**	**E**:52.06±3.79 **C**:51.34±3.76	67/67	**E**:ZSXP powder(10g,TID)+Domperidone(10mg,T ID)+Famotidine(20mg,BID) **C**:Domperidone(10mg,TID)+Famotidine(20mg,BID)	NA	GAS	NR

Diagnostic criteria ①: Rome Criteria; ②: Rome II Criteria; ③: Rome III Criteria;④: Rome IV Criteria; ⑤: Consensus on the diagnosis and treatment of Chinese children with functional dyspepsia(FD); ⑥: Standards of TCM diagnosis and treatment of functional dyspepsia(FD); ⑦: Functional gastroduodenal disorders [47]

NA: unavailable; NR: no record; TER = total effective rate; **E** = experimental group; **C** = control group; **D** = Day; **W** = week; **M** = month; ZSXP = Zhishixiaopi decoction; QD = once a day; BID = twice a day; TID = three times a day

GMFI: Gastric motility function index; GE = Gastric emptying rate, GER(B) = Gastric emptying rate (GE) using a Barium meal method; GER(U) = Gastric emptying rate (GE)using an ultrasound-based method; GHET(T_1/2_) = Gastric half emptying time using an Ultrasonic method (GHET(T1/2)); EM = Esophageal manometry; VNTM = Vagus nerve tone measurement; SAS = SAS Anxiety Scale; SF-36 = SF-36 Quality of Life Scale; SS = Somatostatin; MOT = Motilin, GAS = Gastrin; VIP = Vasoactive peptide; LEP = Leptin

### Meta-analysis results

**Fig 3:**
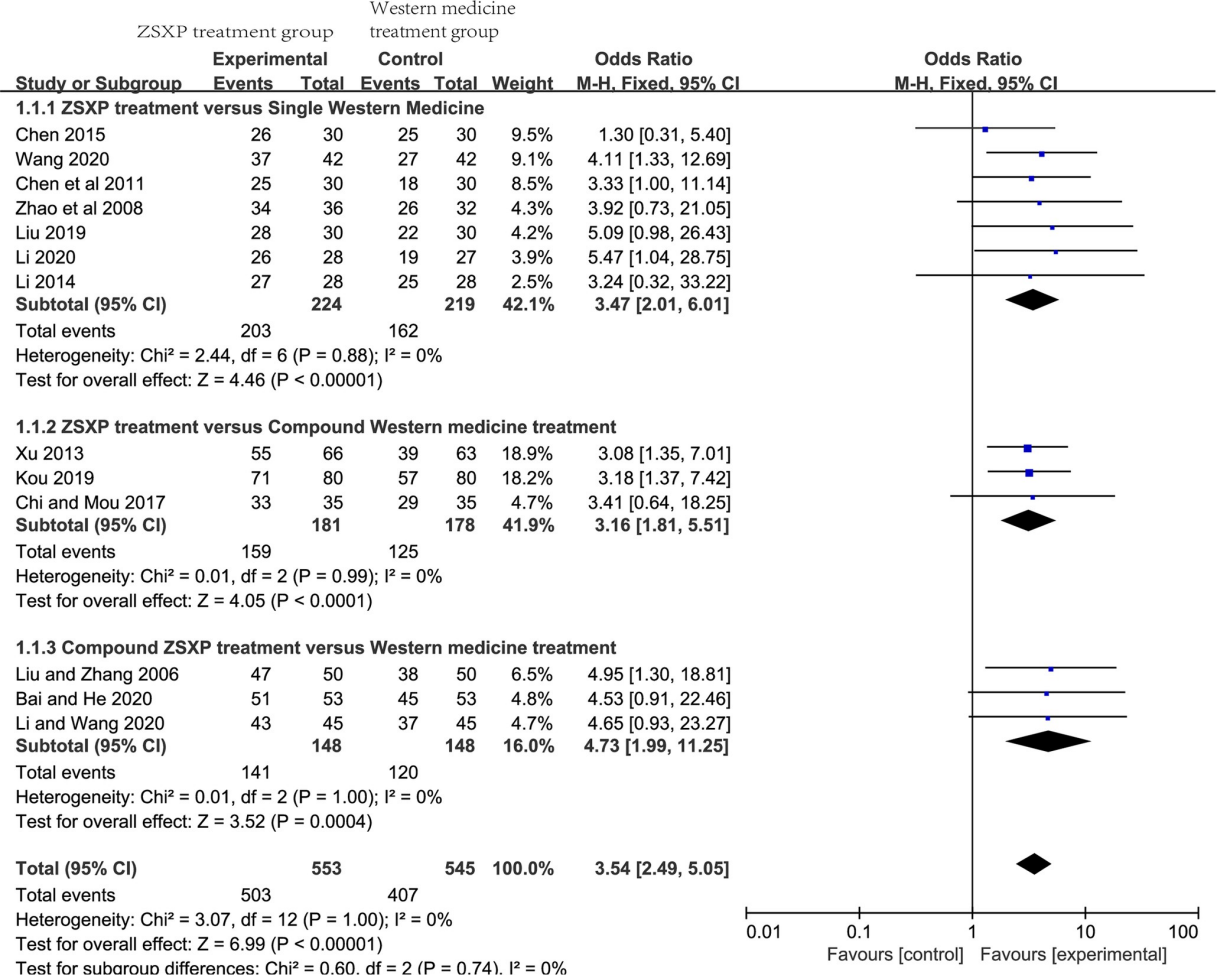
Forest plot (Total effective rate, TER). Forest plots of total effective rate (TER) for comparison of ZSXP treatment between Western medicine treatment.

A meta-analysis was conducted on the thirteen studies [28, 30–34, 37, 39–44] reporting the TER. The heterogeneity test showed no significant heterogeneity among all the studies (*P* = 1.00, *I*^*2*^ = 0%). A fixed-effect model was used. The results suggested that compared with the Western medicine treatment group, the ZSXP treatment group exhibited greater improvement in the TER [OR = 3.54, 95%CI:(2.49, 5.05), Z = 6.99, *P*<0.00001] (Fig 3). At the same time, to avoid the influence of different intervention methods on the analysis results, we conducted subgroup analysis according to different intervention methods and obtained consistent statistical results in each subgroup (Fig 3). We conducted a sensitivity analysis in each subgroup measure by removing the studies with the highest weights one by one. The heterogeneity remained qualitatively consistent for each measure. This indicated that the result of meta-analysis was stable.

**Fig 4:**
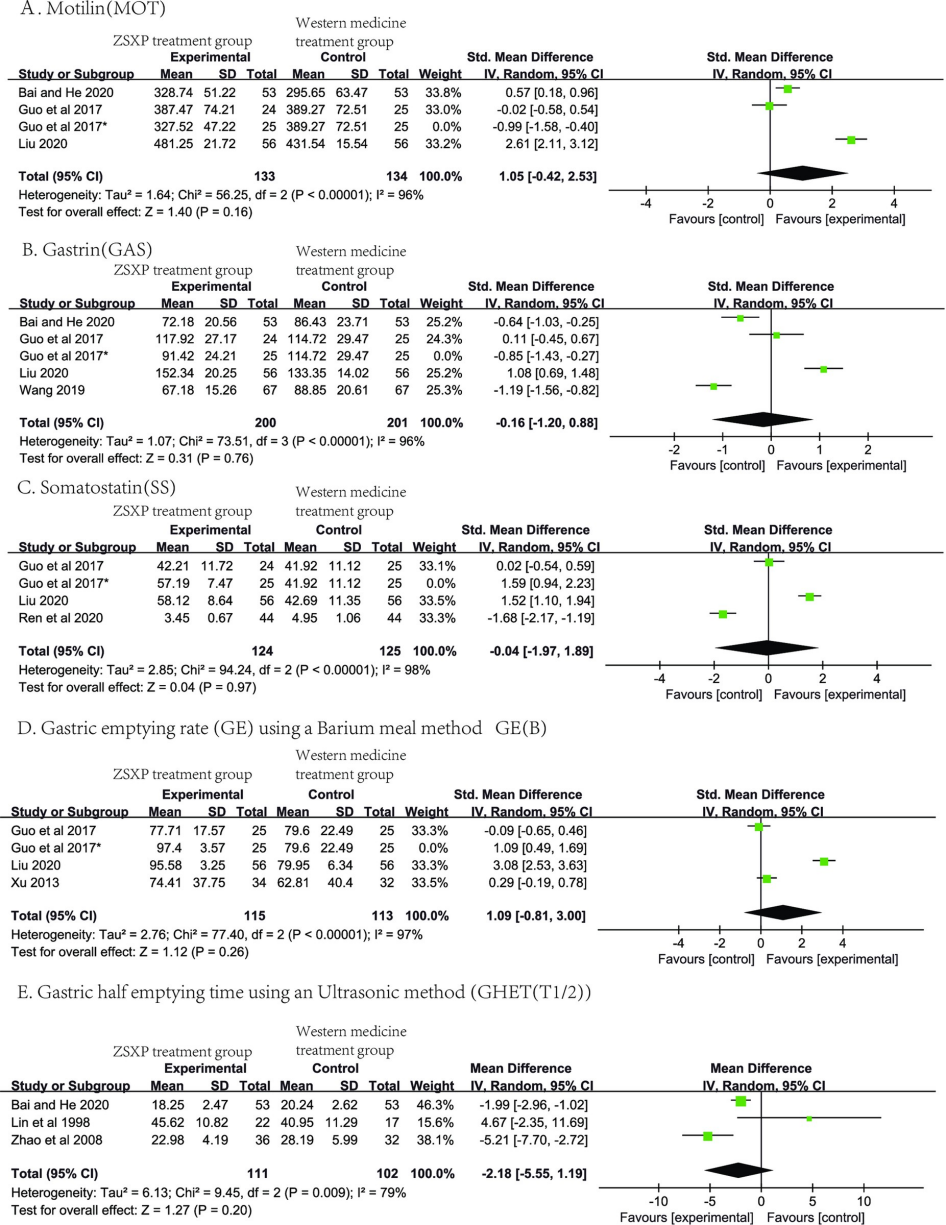
Forest plots of other outcomes. Forest plots of Motilin(**4A**), Gastrin(**4B**), Somatostatin(**4C**), Gastric emptying rate(**4D**), Gastric half emptying time(**4E**) for comparison of ZSXP treatment between Western medicine treatment.

Serum MOT values are recorded in 4 studies [35, 36, 42, 45], but the data of MOT values in a study [36] was not available in the meta-analysis because it used healthy adults as controls. There was significant heterogeneity among the other three studies [35, 42, 45] (*P*<0.00001, *I*^*2*^ = 96%), a random-effect model was used. The meta-analysis suggested that there was no statistical significance in the serum MOT values of FD between the ZSXP treatment group and the Western medicine treatment group [SMD = 1.05, 95%CI:(-0.42, 2.53), *Z* = 1.04, *P* = 0.16] (Fig 4A).

The meta-analysis of the serum GAS values of these four studies [35, 42, 45] suggested that there was no statistical significance between the experimental group and the control group by a random-effect model [SMD = -0.16, 95%CI:(-1.20, 0.88), *Z* = 0.31, *P* = 0.76], with significant heterogeneity (*P*<0.00001, *I*^*2*^ = 96%) (Fig 4B).

For serum SS values, three studies [27, 35, 45] showed significant heterogeneity (*P*<0.00001, *I*^*2*^ = 98%). A following random-effect model and meta-analysis suggested no statistical significance between groups [SMD = -0.04, 95%CI:(-1.97, 1.89), *Z* = 0.04, *P* = 0.97] (Fig 4C).

The data of GER(B) from three studies [35, 37, 45] were included in the meta-analysis. The heterogeneity between studies was significant (*P*<0.00001, *I*^*2*^ = 97%). Following analysis suggested no statistically significant difference in GER(B) between groups by a random-effect model [SMD = 1.09, 95%CI:(-0.81, 3.00), *Z* = 1.12, *P* = 0.26] (Fig 4D). For GHET(T_1/2_), there was significant heterogeneity among the three studies [26, 33, 42] (*P*<0.009, *I*^*2*^ = 79%). Using a random-effects model, the meta-analysis suggested that there was no statistically significant difference between groups [MD = -2.18, 95%CI:(-5.55, 1.19), *Z* = 1.27, *P* = 0.20] (Fig 4E).

It is worth noting that a study (Guo et al., 2017 [35]) has three sets of data and two sets of comparisons. The results of the meta-analysis of serum MOT, GAS and SS values, and GER(B) did not change regardless of whether one or both sets of data were included. In order to avoid inclusion of duplicate data, only one set of comparison data was randomly included in the reported results. We conducted a sensitivity analysis in each of serum MOT, GAS and SS values, GER(B), and GHET(T_1/2_) measures by removing the studies with the highest weights one by one. The heterogeneity remained qualitatively consistent for each measure. This indicated that the result of meta-analysis was stable.

### Publication bias

**Fig 5:**
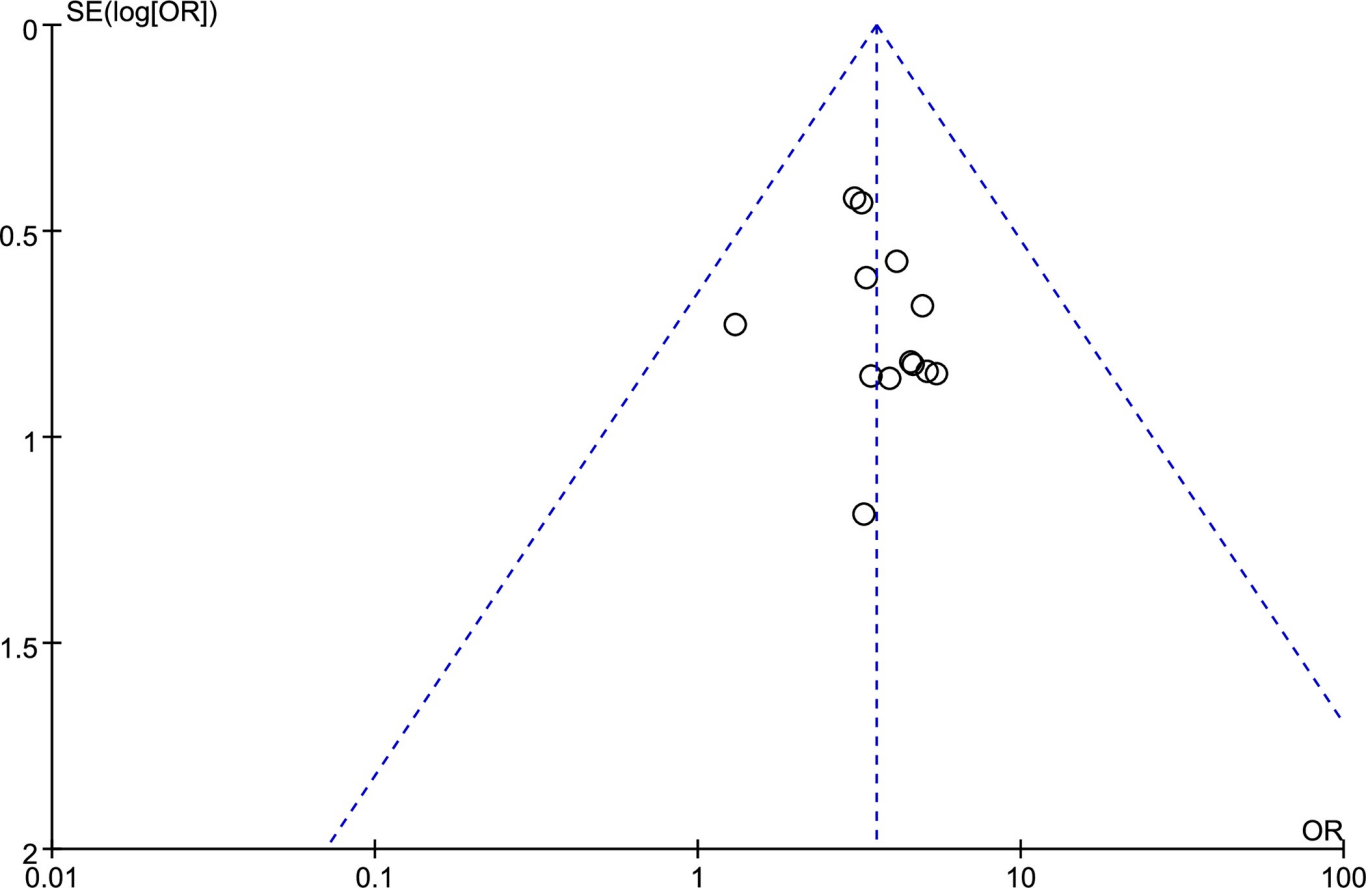
Funnel plot for TER.

The funnel plot for TER was performed including thirteen RCTs (Fig 5). The plot indicated that none of the thirteen points showed obvious asymmetry. In other words, that publication bias was considered small. Since there were too few included studies with serum MOT, GAS and SS values, GER(B) and GHET(T_1/2_) measures, publication bias was unable to be evaluated by funnel plot.

### Adverse events

A total of eleven [28–34, 37, 39, 41, 44] of the 21 included studies recorded adverse events, seven [31–33, 37, 39, 41, 44] of the eleven studies reported no adverse events in either the experimental group or the control group. Four [28–30, 34] of the studies reported adverse events. Adverse events occurred in the control group in three [28, 30, 34] out of the four studies. In one study [29], adverse events occurred in both two groups. The adverse events in the experimental group included dizziness, headache, palpitations, rash and fatigue. Adverse events in the control group included diarrhea, absolute pain, dry mouth, dizziness, fatigue, elevated alanine aminotransferase, lactation phenomenon, headache and skin rash. These data suggest that, compared to Western medicine treatment, ZSXP treatment did not significantly increase the incidences of adverse events (Table 3).

**Table 3:** Adverse event records in each study.

	Experimental group (ZSXP treatment group)		Control group (Western medicine treatment group)	
	Events	Sample size	Events	Sample size
**Chen et al., 2011 [30]**	0	30	NR	30
	—		diarrhea and abdominal pain (n = 5, 16.7%; 5/30) dry mouth (n = 6, 20.0%; 6/30) dizziness and fatigue (n = 8, 26.7%; 8/30) elevated alanine aminotransferase (n = 2, 6.7%; 2/30)	
**Chen, 2015 [28]**	0	30	3	30
	—		dry mouth (n = 1, 3.3%; 1/30) diarrhea (n = 2, 6.7%; 2/30)	
**Li, 2014 [31]**	0	28	0	28
**Liu, 2019 [32]**	0	30	0	30
**Wang, 2020 [33]**	0	42	0	42
**Zhao et al., 2008 [34]**	0	36	1	32
	—		lactation phenomenon (n = 1, 3.1%, 1/32)	
**Xu, 2013 [37]**	0	66	0	63
**Kou, 2019 [39]**	0	80	0	80
**Li, 2020 [41]**	0	28	0	27
**Xu and Zhao, 2018 [29]**	10	134	8	134
	dizziness (n = 3, 2.2%; 3/134) headache (n = 1, 0.7%; 1/134) palpitations (n = 1, 0.7%; 1/134) rash (n = 2, 1.5%; 2/134) fatigue (n = 3, 2.2%; 3/134)		headache (n = 3, 2.2%; 3/134) skin rash (n = 2, 1.5%; 2/134) dry mouth (n = 3, 2.2%; 3/134)	
**Li and Wang, 2020 [44]**	0	56	0	56

## Discussion

The meta-analysis suggested that ZSXP treatment was more effective than Western medicine treatment group in symptom improvement measured by TER while not showing significant increase in adverse events. Prokinetic drugs are the most commonly used prescription for treating FD. A previous meta-analysis of 29 RCTs suggested that prokinetic agents are more effective than placebo [RR = 0.81; 95% CI: (0.74, 0.89)] [48]. Eleven [28, 30–34, 37, 39, 40, 42, 43] out of the thirteen RCTs [28, 30–34, 37, 39–44] included in this meta-analysis use prokinetics as a control condition, suggesting that the better TER of ZSXP than Western medicine was mainly driven by previous research reporting that herbal medicines have shown efficacy comparable to recognized drugs in treating FD [49].

Herbal medicine is a promising treatment for FD as it simultaneously targets multiple pathophysiological mechanisms [50]. In our study, compared to the Western medicine treatment group, ZSXP treatment did not show a significant advantage in serum level of MOT, GAS or SS. The reasons may be as follows. First, MOT and SS are not accurate biological markers to capture FD gastrointestinal motility. Previous studies have shown that the serum levels of SS and MOT did not differ between healthy controls and FD patients [13, 51]. The association of FD symptom relief with serum GAS level is also modest. Some studies suggest that GAS serum level did not predict the response to H2 blocker therapy in FD, even though acid suppressive therapy is often applied to FD as first-line therapy [52]. It is currently unclear to what extent the serum levels of MOT, GAS and SS can reflect the effects of medicine treatment on gastrointestinal function in FD. Thus. the insensitivity of MOT, GAS and SS serum levels to treatment response may lead to no significant difference in these measures between the ZSXP treatment group and the Western medicine treatment group. On the other hand, the reason may be related to too few included studies and the heterogeneity among studies. The heterogeneity among studies was mainly due to inconsistent intervention methods between experimental groups and control groups.

GER is an indicator that can reflect gastrointestinal function. The meta-analysis suggested that compared with Western medicine treatment, ZSXP treatment did not significantly improve GER(B) and GHET(T_1/2_) scores in FD. Several previous studies and meta-analyses have documented the lack of association between the improvement of symptoms and the improvement in GER with prokinetic therapy [16, 53, 54]. Relevant research shows that the severity of symptoms in FD is not correlated to gastric emptying rate [15], Previous data suggested that only up to 25%-30% of FD [16, 17] patients show impaired gastric emptying. These suggest that the association of FD symptoms with GER is modest and the assessment of gastric emptying does not necessarily predict the therapeutic outcome [53]. The results of meta-analysis in GER(B) and GHET(T_1/2_) were also related to the small number and high heterogeneity of the included studies.

Recent studies suggested that single herb in ZSXP can play a role in the gastrointestinal tract functioning. Zhishi can improve colonic motility in rats [55]. The flavonoids of Zhishi can regulate gastrointestinal function [56] and reduce rumen inflammation of Holstein bulls [57]. Houpo also has a regulating effect on the gastrointestinal tract, and its components mainly act as non-competitive muscarinic antagonists in the gastrointestinal tract [58]. Banxia and Huanglian often appear in prescriptions in pairs. This herbal pair is effective in the treatment of Diabetic Gastroparesis [59, 60]. The prescription Banxia Xiexin Decoction composed of this herbal pair is also effective for FD [61]. Sijunzi decoction (SJZ) is included in ZSXP, study suggested that SJZ can promote IEC-6 cell migration and proliferation by activating TLR-2 / My D88 signaling pathway and repairing the injury of the gastrointestinal mucosal barrier [62]. SJZ can improve gastric electrokinesis [63], regulate the levels of MOT, CCK and SS to promote GER in disease model rats [64]. SJZ can rectify digestive disorders in mice [65]. Also, a meta-analysis of RCTs suggested that SJZ is effective for the symptomatic improvement of FD [66]. In FD rats, ZSXP has the neuroprotective effect against autophagy-induced damage and apoptosis occurs by blocking the mTOR pathway in Cort-induced PC12 cells [67]. This may be the potential mechanism of action of ZSXP on FD. Unfortunately, due to the complexity of herbal medicine research itself and FD pathophysiology, the mode of action of ZSXP remains underexplored and may act through multiple pathophysiological mechanisms.

FD is a type of psychological co-morbidity [2, 5]. Houpo has anti-anxiety and anti-depressant effects in a single-drug study of ZSXP [58]. At present, there are few studies on the psychological effects of ZSXP for FD. More psychological assessment is needed in this research direction.

There are also some limitations to this study. First of all, the quality of the included studies is moderate. In particular, there is a lack of high-quality, multi-center, and large-scale studies. The single herb dosage of ZSXP is not recorded or may differ among the included studies, which would affect the stability of the results. Second, the included studies have little information about the psychological assessment of FD patients. As a result, we were unable to assess the effect of ZSXP on the psychological states of FD patients. Therefore, more research is needed to fully confirm the efficacy and safety of ZSXP for treating FD.

On the whole, the meta-analysis evaluated the efficacy and safety of ZSXP for FD for the first time. The result of the study could provide clinicians with data support for decision-making and provide patients with another alternative treatment to choose from. Available data suggested that, compared with Western medicine treatment, ZSXP treatment is more effective for FD without obvious adverse events, however, did not show superiority in MOT, GAS and SS serum levels, GER(B) and GHET(T1/2) of FD. Due to the complexity of herbal medicine research itself and FD pathophysiology, the mode of action of ZSXP remains underexplored and may act through multiple pathophysiological mechanisms. More laboratory tests that can sensitively respond to FD treatment need to be further identified. More psychological assessment is needed in the FD research direction. More high-quality, multi-center, and large-sample RCTs are needed to fully confirm the efficacy and safety of ZSXP for treating FD.

## Conclusion

The meta-analysis suggests that ZSXP treatment is a relatively effective and safe traditional Chinese medicine prescription and could be used for FD treatment. Considering the limitations of this study, the conclusion needs to be further confirmed by high-quality, multi-center, and large-sample RCTs.

## Supporting information

S1 Checklist The PRISMA 2020 checklist. (DOCX)

S1 File Search strategy. (PDF)

S2 File Minimal data set. (DOCX)
