# Supplementary material for: Efficacy and safety of Zhishixiaopi decoction in functional dyspepsia: A meta-analysis of randomized controlled trials
Source: PLoS One. 2024 May 29;19(5):e0301686. doi: 10.1371/journal.pone.0301686 (PMC11135732; doi:10.1371/journal.pone.0301686)
Supplement: S1 File — (PDF) [file pone.0301686.s002.pdf]

Efficacy and Safety of Modified Zhishixiaopi Decoction in Functional Dyspepsia: A meta-analysis of randomized controlled trials

Xiankun Zhao[1], Xinyu Cheng[2], Jing Ye[3], Jiaqing Ren[4], Bin Li[1], Dongmei Tan[1], Tangshan Li[1], Kai Zhou[1], Jing Pu[1], Xia Luo[1], Yong Feng[1,*]

S1 File. Search strategy.

Appendix 1. PubMed search strategy

#1 indigestion*
#2 Intestin* OR Digest* OR Gastr* OR gut OR epigastr* OR stomach*
#3 #1 AND #2
#4 dyspepsia*
#5 epigastric [tiab] AND pain [tiab]
#6 epigastric [tiab] AND burn* [tiab]
#7 (disturbance* OR disorder* OR difficult* OR dysfunction* OR disease* OR impair* OR condition* OR abnormal* OR illness* OR patholog* OR discomfort* OR hazard* OR damage* OR injur* OR irritab* OR pain* OR distress* OR burning) AND postprandial*
#8 #3 OR #4 OR #5 OR #6 OR #7
#9 Herbal medicine [MeSH Terms]
#10 Plants, medicinal [MeSH Terms]
#11 Medicine, traditional [MeSH Terms]
#12 Drugs, Chinese herbal [MeSH Terms]
#13 Herb* [tiab]
#14 Plant [tiab] OR plants [tiab]
#15 Phytomedicine [tiab]
#16 Botanical [tiab]
#17 (Traditional [tiab] OR Chinese [tiab] OR herbal [tiab]) AND medicine[tiab]
#18 (Oriental [tiab] OR Chinese [tiab]) AND tradition* [tiab]
#19 #9 OR #10 OR #11 OR #12 OR #13 OR #14 OR #15 OR #16 OR #17 OR #18
#20 Zhishixiaopi* OR Zhishi Xiaopi* OR Zhi shi xiao pi* OR Kaiwei*

#21 Randomized controlled trial [pt]
#22 Controlled clinical trial [pt]
#23 Randomized [tiab]
#24 Randomly [tiab]
#25 Trial [tiab]
#26 #21 OR #22 OR #23 OR #24 OR #25
#27 #8 AND #19 AND #20 AND #26

Appendix 2. Ovid search strategy

Embase <1974 to 2021 December 03>
Ovid MEDLINE(R) <1946 to December Week 1 2021>

1 indigestion*.af.
2 (Intestin* or Digest* or Gastr* or gut or epigastr* or stomach*).af.
3 1 and 2
4 dyspepsia*.af.
5 (epigastric and pain).ab,ti.
6 (epigastric and burn*).ab,ti.
7 (disturbance* or disorder* or difficult* or dysfunction* or disease* or impair* or condition* or abnormal* or illness* or patholog* or discomfort* or hazard* or damage* or injur* or irritab* or pain* or distress* or burning).af.
8 postprandial*.af.
9 7 and 8
10 3 or 4 or 5 or 6 or 9
11 (Herbal medicine or Plants, medicinal or Medicine, traditional or Drugs, Chinese herbal).af.
12 (Herb* or Plant or plants or Phytomedicine or Botanical).ab,kf,kw,ti.
13 11 or 12
14 (Zhishixiaopi* or Zhishi Xiaopi* or Zhi shi xiao pi* or Kaiwei* or Shixiaowan).af.
15 Randomized controlled trial.pt.
16 Controlled clinical trial.pt.
17 (Randomized or Randomly or Trial).ab,ti.
18 15 or 16 or 17
19 10 and 13 and 14 and 18

Appendix 3. The Cochrane Library search strategy

#1 (Intestin* OR Digest* OR Gastr* OR gut OR epigastr* OR stomach*) AND (indigestion*)
#2 dyspepsia*
#3 (epigastric AND pain):ti,ab,kw
#4 (epigastric AND burn*):ti,ab,kw

#5(disturbance* OR disorder* OR difficult* OR dysfunction* OR disease* OR impair* OR condition* OR abnormal* OR illness* OR patholog* OR discomfort* OR hazard* OR damage* OR injur* OR irritab* OR pain* OR distress* OR burning) AND (postprandial*)
#6 #1 OR #2 OR #3 OR #4 OR #5
#7 MeSH descriptor: [Herbal Medicine] explode all trees
#8 MeSH descriptor: [Medicine, Traditional] explode all trees
#9 MeSH descriptor: [Drugs, Chinese Herbal] explode all trees
#10 MeSH descriptor: [Plants, Medicinal] explode all trees
#11 (Herb* OR Plant OR plants OR Phytomedicine OR Botanical):ti,ab,kw
#12 ((Traditional OR Chinese OR herbal) AND medicine):ti,ab,kw
#13 ((Oriental OR Chinese) AND tradition*):ti,ab,kw
#14 #7 OR #8 OR #9 OR #10 OR #11 OR #12 OR #13
#15 (Zhishixiaopi* OR Zhishi Xiaopi* OR Zhi shi xiao pi* OR Kaiwei* OR Shixiaowan)
#16 (Randomized controlled trial):pt
#17 (Controlled clinical trial):pt
#18 (Randomized):ti,ab,kw
#19 (Randomly):ti,ab,kw
#20 (Trial):ti,ab,kw
#21 #16 OR #17 OR #18 OR #19 OR #20
#22 #6 AND #14 AND #15 AND #21

Appendix 4. Web of Science search strategy

1 TS=(indigestion*)
2 TS=(Intestin* OR Digest* OR Gastr* OR gut OR epigastr* OR stomach*)
3 #1 AND #2
4 TS=(dyspepsia*)
5 TI=(epigastric AND pain) OR AB=(epigastric AND pain) OR AK=(epigastric AND pain)
6 TI=(epigastric AND burn*) OR AB=(epigastric AND burn*) OR AK=(epigastric AND burn*)
7 TS=((disturbance* OR disorder* OR difficult* OR dysfunction* OR disease* OR impair* OR condition* OR abnormal* OR illness* OR patholog* OR discomfort* OR hazard* OR damage* OR injur* OR irritab* OR pain* OR distress* OR burning) AND (postprandial*))
8 #3 OR #4 OR #5 OR #6 OR #7
9 TS=(Herbal Medicine)
10 TS=(Medicine, Traditional)
11 TS=(Drugs, Chinese Herbal)
12 TS=(Plants, Medicinal)
13 TS=(Herb* OR Plant OR plants OR Phytomedicine OR Botanical)
14 TS=((Traditional OR Chinese OR herbal) AND medicine)
15 TS=((Oriental OR Chinese) AND tradition*)
16 #9 OR #10 OR #11 OR #12 OR #13 OR #14 OR #15
17 (((TS=(Zhishixiaopi*)) OR TS=(Zhishi Xiaopi*)) OR TS=(Kaiwei*)) OR TS=(Shixiaowan)
18 ((TS=(Randomized)) OR TS=(Randomly)) OR TS=(Trial)

19 (TS=(Randomized controlled trial)) OR TS=(Controlled clinical trial)
20 #18 OR #19
21 #8 AND #16 AND #17 AND #20

Appendix 5. CBM search strategy (in Chinese)

#1 "Lin chuang shi yan" [Weighted: expanded]
#2 Lin chuang shi yan OR Lin chuang guan cha OR Lin chuang liao xiao OR Lin chuang xiao guo OR Lin chuang yan jiu OR Lin chuang ping jia OR Lin chuang ping gu
#3 Dan mang OR Shuang mang OR San mang OR Mang fa OR An wei ji OR Sui ji OR Yan jiu she ji
#4 "Sui ji dui zhao shi yan"[Weighted: expanded] OR "Sui ji fen pei" [Weighted: expanded] OR "Shuang mang fa" [Weighted: expanded] OR "Dan mang fa" [Weighted: expanded]
#5 #1 OR #2 OR #3 OR #4
#6 "Ping jia yan jiu" [Weighted: expanded] OR "Sui fang yan jiu" [Weighted: expanded] OR "Qian zhan xing yan jiu" [Weighted: expanded]
#7 "Dui zhao"[Chinese title: intelligence] OR "Dui bi"[Chinese title: intelligence] OR "Bi jiao"[Chinese title: intelligence] OR "Zi yuan"[Chinese title: intelligence]
#8 "Dui zhao"[Abstract: intelligence] OR "Dui bi"[Abstract: intelligence] OR "Bi jiao"[Abstract: intelligence] OR "Zi yuan"[Abstract: intelligence]
#9 "Dui zhao"[keywords: intelligence] OR "Dui bi"[keywords: intelligence] OR "Bi jiao"[keywords: intelligence] OR "Zi yuan"[keywords: intelligence]
#10 #6 OR #7 OR #8 OR #9
#11 #5 OR #10
#12 "Dyspepsia"[All fields: intelligence] OR "Non ulcer dyspepsia"[All fields: intelligence] OR "Functional dyspepsia"[All fields: intelligence]
#13 "Shao zhuo"[All fields: intelligence] OR "Zhuo re"[All fields: intelligence] OR "Shi yu bu zhen"[All fields: intelligence] OR "Shang fu bu shi"[All fields: intelligence] OR "Shang fu tong"[All fields: intelligence] OR "Fu zhang"[All fields: intelligence] OR "Zao bao"[All fields: intelligence]OR "Ai qi" [All fields: intelligence]
#14 #12 OR #13
#15 #11 AND #14
#16 "ZhishiXiaopi%" [All fields: intelligence] OR "Kaiweifang" [All fields: intelligence] OR "Kaiweitang" [All fields: intelligence]
#17 #15 AND #16

Appendix 6. CNKI search strategy (in Chinese)

1) SU=Sui ji fen pei OR SU=Sui ji OR SU=Shuang mang OR SU=Dan mang OR SU=San mang OR SU=An wei ji
2) SU=Sui ji dui zhao shi yan OR SU=Lin chuang shi yan OR SU=Lin chuang guan cha OR SU=Lin chuang liao xiao OR SU=Lin chuang xiao guo OR SU=Lin chuang yan jiu OR SU=Lin

chuang ping jia OR SU=Lin chuang ping gu
3) TI=Dui zhao OR TI=Dui bi OR TI=Bi jiao OR TI=Zi yuan
4) AB=Dui zhao OR AB= Dui bi OR AB= Bi jiao OR AB= Zi yuan
5) KY= Dui zhao OR KY= Dui bi OR KY= Bi jiao OR KY= Zi yuan
6) (#1) OR (#2) OR (#3) OR (#4) OR (#5)
7) SU=dyspepsia OR SU=non ulcer dyspepsia OR SU=functional dyspepsia OR SU=Shao zhuo OR SU=Zhuo re OR SU=Shi yu bu zhen OR SU=Shang fu bu shi OR SU=Shang fu tong OR SU=Fu zhang OR SU=Zao bao OR SU=Ai qi
8) (#6) AND (#7)
9) SU=Zhishi Xiaopi% OR SU=Kaiweifang OR SU=Kaiweitang
10) (#8) AND (#9)
SU=Subject, TI=Title, AB=Abstract, KY= keywords

Appendix 7. VIP search strategy (in Chinese)

1) M=Sui ji fen pei OR M=Sui ji OR M=Shuang mang OR M=Dan mang OR M=San mang OR M=An wei ji
2) M=Sui ji dui zhao shi yan OR M=Lin chuang shi yan OR M=Lin chuang guan cha OR M=Lin chuang liao xiao OR M=Lin chuang xiao guo OR M=Lin chuang yan jiu OR M=Lin chuang ping jia OR M=Lin chuang ping gu
3) T=Dui zhao OR T=Dui bi OR T=BI jiao OR T=Zi yuan
4) R=Dui zhao OR R=Dui bi OR R=BI jiao OR R=Zi yuan
5) K=Dui zhao OR K=Dui bi OR K=Bi jiao OR K=Zi yuan
6) (#1) OR (#2) OR (#3) OR (#4) OR (#5)
7) M=dyspepsia OR M=non ulcer dyspepsia OR M=functional dyspepsia OR M=Shao zhuo OR M=Zhuo re OR M=Shi yu bu zhen OR M=Shang fu bu shi OR M=Shang fu tong OR M=Fu zhang OR M=Zao bao OR M=Ai qi
8) (#6) AND (#7)
9) M=Zhishixiaopi% OR M=Kaiweifang OR M=Kaiweitang
10) (#8) AND (#9)
T=Title R= Abstract K= keywords

Appendix 8. WanFang Data search strategy (in Chinese)

1) Title or keywords:(Sui ji fen pei OR Sui ji OR Shuang mang OR Dan mang OR San mang OR An wei ji)
2) Title or keywords:(Sui ji dui zhao shi yan OR Lin chuang shi yan OR Lin chuang guan cha OR Lin chuang liao xiao OR Lin chuang xiao guo OR Lin chuang yan jiu OR Lin chuang ping jia OR Lin chuang ping gu)
3) Title:(Dui zhao OR Dui bi OR Bi jiao OR Zi yuan)
4) Abstrac: (Dui zhao OR Dui bi OR Bi jiao OR Zi yuan)
5) keyword: (Dui zhao OR Dui bi OR Bi jiao OR Zi yuan)

6) (#1) OR (#2) OR (#3) OR (#4) OR (#5)

7) Title or keyword:(dyspepsia OR non ulcer dyspepsia OR functional dyspepsia OR Shao zhuo OR Zhuo re OR Shi yu bu zhen OR Shang fu bu shi OR Shang fu tong OR Fu zhang OR Zao bao OR Ai qi)

8) (#6) AND (#7)

9) Title or keyword:(Zhishixiaopi% OR Kaiweifang OR Kaiweitang)

10) (#8) AND (#9)
