# Supplementary material for: Efficacy and safety of Zhishixiaopi decoction in functional dyspepsia: A meta-analysis of randomized controlled trials
Source: PLoS One. 2024 May 29;19(5):e0301686. doi: 10.1371/journal.pone.0301686 (PMC11135732; doi:10.1371/journal.pone.0301686)
Supplement: S2 File — (DOCX) [file pone.0301686.s003.docx]

*Supporting Information*

*Minimal data set*

**Cohen's Kappa**

Cohen's Kappa was used to assess the concordance between the two independent reviewers.

|  | Positive  (Researchers A) | Negative (Researchers A) | Total |
| --- | --- | --- | --- |
| Positive (Researchers B) | 21($n11$) | 3($n12$) | 24($n1.$) |
| Negative (Researchers B) | 1($n21$) | 316($n22$) | 317($n2.$) |
| Total | 22($n.1$) | 319($n.2$) | 341($n11+n12+n21+n22$) |

$$p_{0}=\frac{n11+n22}{n11+n12+n21+n22}=\frac{337}{341}\approx0.988$$

$$Pⅇ=\frac{n.1*n1.+n.2*n2.}{{(n11+n12+n21+n22)}^{2}}=\frac{22*24+319*317}{{(21+3+1+316)}^{2}}=\frac{101651}{116281}\approx0.874$$

$$k=\frac{p_{0}-p_{ⅇ}}{1-p_{e}}=1-\frac{1-P_{0}}{1-p_{e}}=\frac{0.114}{0.126}\approx0.905$$

| $k$ | **Interpretation** |
| --- | --- |
| <0 | Poor agreement |
| 0.0-0.20 | Slight agreement |
| 0.21-0.40 | Fair agreement |
| 0.41-0.60 | Moderate agreement |
| 0.61-0.80 | Substantial agreement |
| 0.81-1.0 | Almost perfect agreement |

Total effective rate (TER)

| **Table 2 Characteristics of the included studies in meta-analysis.** | | | | | | | | |
| --- | --- | --- | --- | --- | --- | --- | --- | --- |
| Study | Diagnostic criteria | Treatment  Duration | Age (years±SD) by group | Sample Size | Interventions | Outcome measures | | Adverse event |
|  |  |  |  |  |  | Symptom improvement (TER) | Others |  |
| **Chen et al., 2011 [30]** | ② | 4 **W** | **E**:42.30±6.23  **C**:41.20±6.41 | 30/30 | **E**:ZSXP(BID)  **C**:Mosapride(5mg, TID) | **E**:83.33%(25/30)  **C**:60.00%(18/30) | NR | Included |
| **Chen, 2015 [28]** | ③ | 15 **D** | **E**:48.63±9.66  **C**:47.26±9.23 | 30/30 | **E**:ZSXP(BID)  **C**:Mosapride(5mg, TID) | **E**:86.67%(26/30)  **C**:83.33%(25/30) | NR | Included |
| **Li, 2014 [31]** | ③ | 4 **W** | **E**:42.9±13.5  **C**:42.7±12.3 | 28/28 | **E**:ZSXP(BID)  **C**:Mosapride(5mg, TID) | **E**:96.43%(27/28)  **C**:89.29%(25/28) | NR | Included |
| **Liu, 2019 [32]** | ④ | 4 **W** | **E**:45.40±10.13  **C**:44.63±10.58 | 30/30 | **E**:ZSXP(BID)  **C**:Mosapride(5mg, TID) | **E**:93.33%(28/30)  **C**:73.33%(22/30) | NR | Included |
| **Wang, 2020 [33]** | ④ | 4 **W** | **E**:45.60±11.17  **C**:45.69±10.32 | 42/42 | **E**:ZSXP(BID)  C:Mosapride(5mg, TID) | **E**:88.09%(37/42)  **C**:64.29%(27/42) | NR | Included |
| **Zhao et al., 2008 [34]** | ③ | 2 **W** | **E**:18-65  **C**:20-65 | 36/32 | **E**:ZSXP(TID)  **C**:Domperidone(10mg,TID) | **E**:94.46%(34/36)  **C**:81.25%(26/32) | GHET( T_1/2_) | Included |
| **Guo et al., 2017 [35]** | ③ | 2 **W** | **E1**:51.2±6.14  **E2**:52.9±4.12  **C**:52.2±6.29 | 24/25/25 | **E1**:ZSXP(BID)  **E2**:ZSXP(BID)+Mosapride(5 mg, TID)  **C**:Mosapride(5mg, TID) | NA | MOT/GAS/  SS/GER(B) | NR |
| **Lin et al., 1998 [26]** | ① | 4 **W** | **E**:45.1±9.67  **C**:48.5±12.9 | 27/24 | **E**:ZSXP(TID)  **C**:Cisapride(5mg,TID) | NA | GHET(T_1/2_) | NR |
| **Dou et al., 2006 [36]** | ⑦ | 4 **W** | **E1**:39.76±12.12  **E2**:37.27±10.29  **E3**:35.22±11.35  **C**:36.30±11.29 | 66/  46/  45/  42 | **E1**:ZSXP(BID)Chinese Herbal Pieces Group  **E2**:ZSXP(BID)Combined decoction granule Group  **E3**:ZSXP(BID)Separated decoction granule group  **C**:Cisapride(5mg,TID) | NA | MOT/ EM/  VNTM | NR |
| **Xu, 2013 [37]** | ③ | 4 **W** | **E**:40.68±11.41  **C**:40.31±12.78 | 66/63 | **E**:ZSXP(BID)  **C**:Domperidone(10mg,TID)+Magnesium aluminum carbonate (1.0g,TID) | **E**:83.33%(55/66)  **C**:61.90%(39/63) | GER(B) | Included |
| **Zhang, 2013 [38]** | ③ | 4 **W** | E:38.80±11.79  C:38.86±12.31 | 66/63 | **E**:ZSXP(BID)  **C**:Domperidone(10mg,TID)+Magnesium aluminum carbonate (1.0g,TID) | NA | VIP, LEP | NR |
| **Kou, 2019 [39]** | ① | 1 **M** | **E**:42.85±6.04  **C**:41.14±5.74 | 80/80 | **E**:ZSXP(BID)  **C**:Domperidone(10mg,TID)+Omeprazole(20mg,BID) | **E**:88.75%(71/80)  **C**:71.25%(57/80) | SF-36/SAS | Included |
| **Chi and Mou,2017 [40]** | ③ | 4 **W** | **E**:53.6±6.25  **C**:52.3±7.12 | 35/35 | **E**:ZSXP(BID)  **C**:Pantoprazole(40mg, QD)+Mosapride(5mg, TID) | **E**:94.29%(33/35)  **C**:82.86%(29/35) | NR | NR |
| **Li, 2020 [41]** | ④ | 4 **W** | **E**:47.86±9.01  **C**:48.78±9.69 | 28/27 | **E**:ZSXP(BID)  **C**:Azintamide(150mg, TID) | **E**:92.9%(26/28)  **C**:70.4%(19/27) | NR | Included |
| **Xu and Zhao, 2018 [29]** | ⑤ | 2 **W** | **E**:9.17±1.82  **C**:9.23±1.84 | 134/134 | **E**:ZSXP pills(0.1g/Kg,TID) +Mosapride(5mg, TID)  **C**:Mosapride(5mg, TID) | NA | GER(U) | Included |
| **Bai and He,2020 [42]** | ④ | 2 **W** | **E**:45.16±3.51  **C**:44.25±3.68 | 53/53 | **E**:ZSXP(NR)+Mosapride(5 mg, TID)  **C**:Mosapride(5mg, TID) | **E**:96.23%(51/53)  **C**:84.91%(45/53) | MOT/GAS/  GMFI/SF-36/ GHET( T_1/2_) | NR |
| **Liu and Zhang, 2006 [43]** | ⑥ | 4 **W** | **E**:57±23.5  **C**:55±25.1 | 50/50 | **E**:ZSXP(QD)+ Mosapride(5mg, TID)  **C**:Mosapride(5mg, TID) | **E**:94.00%(47/50)  **C**:76.00%(38/50) | NR | NR |
| **Li and Wang,2020 [44]** | ④ | 4 **W** | **E**:42.5±3.1  **C**:41.2±3.1 | 45/45 | **E**:ZSXP(BID)+Azintamide(150mg, TID)  **C**:Azintamide(150mg, TID) | **E**:95.6%(43/45)  **C**:82.2%(37/45) | NR | NR |
| **Liu, 2020 [45]** | ③ | 4 **W** | **E**:54.01±6.25  **C**:53.12±6.24 | 56/56 | **E**:ZSXP(BID)+Domperidone(10mg,TID)+Omeprazole(20mg, BID)  **C**:Domperidone(10mg,TID)+Omeprazole(20mg, BID) | NA | MOT/GAS/  SS/GER(B) | Included |
| **Ren et al.,2020 [27]** | ③ | 2 **M** | **E**:33.2±8.5  **C**:32.1±8.2 | 44/44 | **E**:ZSXP pills(6g,TID)+Trimebutine(0.2g TID)  **C**:Trimebutine(0.2g TID) | NA | HP/5-HT/  SS/NO | NR |
| **Wang, 2019 [46]** | ③ | 1 **M** | **E**:52.06±3.79  **C**:51.34±3.76 | 67/67 | **E**:ZSXP powder(10g,TID)+Domperidone(10mg,T  ID)+Famotidine(20mg,BID)  **C**:Domperidone(10mg,TID)+Famotidine(20mg,BID) | NA | GAS | NR |
| Diagnostic criteria ①: Rome Criteria; ②: Rome II Criteria; ③: Rome III Criteria;④: Rome IV Criteria; ⑤: Consensus on the diagnosis and treatment of Chinese children with functional dyspepsia(FD); ⑥: Standards of TCM diagnosis and treatment of functional dyspepsia(FD); ⑦: Functional gastroduodenal disorders [47] ;  NA: unavailable; NR: no record; TER=total effective rate; **E**=experimental group; **C**=control group; **D**=Day; **W**=week; **M**=month; ZSXP=Zhishixiaopi decoction; QD=once a day; BID=twice a day; TID=three times a day;  GMFI: Gastric motility function index; GE=Gastric emptying rate, GER(B)= Gastric emptying rate (GE) using a Barium meal method; GER(U)=Gastric emptying rate (GE)using an ultrasound-based method; GHET(T_1/2_)= Gastric half emptying time using an Ultrasonic method (GHET(T1/2)); EM=Esophageal manometry; VNTM=Vagus nerve tone measurement; SAS=SAS Anxiety Scale; SF-36=SF-36 Quality of Life Scale; SS=Somatostatin; MOT=Motilin, GAS=Gastrin; VIP=Vasoactive peptide; LEP=Leptin; | | | | | | | | |
